# Effectiveness of Telemedicine-Delivered Carbohydrate-Counting Interventions in Patients With Type 1 Diabetes: Systematic Review and Meta-Analysis

**DOI:** 10.2196/59579

**Published:** 2025-04-10

**Authors:** Yang Li, Yue Yang, Xiaoqin Liu, Xinting Zhang, Fei Li

**Affiliations:** 1 Department of Endocrinology and Metabolism The First Hospital of Jilin University Changchun, Jilin China; 2 School of Nursing Jilin University Changchun China; 3 Department of Neurotrauma Surgery The First Hospital of Jilin University Changchun China

**Keywords:** type 1 diabetes, hemoglobin A_1c_, telemedicine, carbohydrate counting, review

## Abstract

**Background:**

Type 1 diabetes mellitus (T1DM) significantly affects patients’ quality of life and can be life-threatening, necessitating improved monitoring strategies. Telemedicine, which leverages telecommunications technologies to deliver health care services and expertise, has the potential to enhance T1DM management. However, its effectiveness remains to be fully established.

**Objective:**

This study aims to evaluate the effectiveness of various telemedicine-based carbohydrate-counting (CC) interventions in patients with T1DM.

**Methods:**

This systematic review and meta-analysis searched 5 databases—PubMed, Web of Science, CINAHL, Embase, and Cochrane—as well as reference lists of retrieved articles on September 26, 2024, for randomized controlled trials (RCTs) assessing the effectiveness of telemedicine-based CC interventions in reducing glycated hemoglobin A_1c_ (HbA_1c_) levels in patients with T1DM.

**Results:**

From 3612 citations, we identified 18 eligible RCTs (n=1627) from 14 regions for inclusion in the meta-analysis. Participants in the telemedicine intervention group experienced a 0.35% reduction in HbA_1c_ levels (95% CI –0.54 to –0.16) compared with the control group. A total of 13 studies used smartphone apps, 4 used connected and wearable glucometers, and 3 delivered the intervention through web-based systems. Significant reductions in HbA_1c_ were observed across smartphone apps (–0.36%, 95% CI –0.63% to –0.09%), connected and wearable glucometers (–0.35%, 95% CI –0.56% to –0.14%), and web-based systems (–0.36%, 95% CI –0.71% to –0.02%). Considerable heterogeneity was noted (I2=81%, *P*<.001). Telemedicine-based CC interventions also increased time in range by 9.59% (95% CI 6.50%-12.67%). However, evidence regarding treatment satisfaction, total daily insulin dose, and hypoglycemia remains inconclusive. Subgroup analysis showed that telemedicine platform variety did not significantly contribute to heterogeneity, while meta-regression indicated that the impact on HbA_1c_ was most pronounced in trials conducted in Asia.

**Conclusions:**

Compared with usual care, telemedicine-delivered CC interventions improved HbA_1c_ and time in range but did not significantly impact other clinically relevant outcomes in patients with T1DM. High-quality, large-scale RCTs are needed to draw definitive conclusions. These findings provide health care professionals with updated evidence on the role of telemedicine in glycemic control for patients with T1DM.

**Trial Registration:**

PROSPERO CRD42024523025; https://www.crd.york.ac.uk/PROSPERO/view/CRD42024523025

## Introduction

Type 1 diabetes mellitus (T1DM) is a chronic autoimmune disease characterized by absolute insulin deficiency due to the immune-mediated destruction of pancreatic β-cells, resulting in hyperglycemia [1,2]. T1DM accounts for 5%-10% of all diabetes cases [3], and its global incidence is rising dramatically [4]. Patients with T1DM require lifelong insulin therapy to maintain blood glucose (BG) levels within recommended ranges and to reduce the risk of both acute and long-term complications [5].

The Academy of Nutrition and Dietetic Sciences has shown that for patients with T1DM, a pivotal element of management is carbohydrate counting (CC) to determine the appropriate preprandial insulin dosage [6]. Ideally, patients should learn to calculate their carbohydrate intake and adjust mealtime insulin doses accordingly [7]. However, CC is considered one of the most onerous tasks in T1DM care [8]. As the effectiveness of CC may be limited by patient adherence and the inability to estimate carbohydrate content accurately, its assessment is often imprecise [9].

The issue of suboptimal glycemic control among patients with T1DM is partly attributed to the complexities of accurately calculating mealtime insulin doses [10]. Furthermore, regular consultations with a specialist are necessary. The intensive and ongoing need for treatment imposes a significant burden, negatively affecting the quality of life of both patients and their families [11]. Telemedicine (TM) may help address these challenges [10].

TM refers to the remote delivery of clinical services through electronic information and telecommunication technologies. It is utilized across a wide range of health care services, including health assessment, diagnosis, intervention, consultation, supervision, and access to information [12]. The American Telemedicine Association defines TM as the use of medical information exchanged from one site to another via electronic communication to improve patients’ clinical health. This includes a growing number of applications and services that utilize 2-way video, smartphones, wireless tools, and other telecommunication technologies [13]. TM can be classified based on the communication method (text, video, or audio), communication time (synchronous or asynchronous), the purpose of the consultation (initial consultation or follow-up consultation), and participants in the remote consultation (patient-to-doctor, caregiver-to-doctor, doctor-to-doctor, or health care worker-to-doctor) [14].

In chronic disease management, particularly diabetes, the integration of TM technology with medical professionals has yielded remarkable results [15]. The American Diabetes Association (ADA 2022) states that TM is a growing field that may improve access to care for people with diabetes [16]. With advancements in technology, TM has evolved beyond simple phone calls and video consultations to include applications such as augmented reality, virtual reality, and artificial intelligence [17]. The incorporation of these emerging technologies not only broadens the definition of TM but also presents new opportunities and challenges for managing chronic diseases such as diabetes. A wide range of new technologies is expected to help alleviate the burden of T1DM. While the potential and feasibility of technology-based approaches have been well established, their effectiveness remains unclear.

Upon reviewing the relevant literature, we found no systematic reviews or meta-analyses evaluating the efficacy of various TM approaches in CC interventions for glycemic control among patients with T1DM. Current evidence suggests that CC is an effective method for lowering HbA_1c_, and patients with T1DM are encouraged to use CC rather than alternative approaches [9]. However, no conclusive evidence is available regarding the effectiveness of TM-delivered CC interventions for T1DM management. This study aimed to conduct a systematic review and quantitative synthesis of randomized controlled trials (RCTs) to assess the effectiveness of a TM-delivered CC intervention in patients with T1DM. The findings may contribute to the development of new approaches to improve the quality of life of patients with T1DM.

## Methods

### Overview

The evaluation protocol was prospectively registered in PROSPERO (International Prospective Register of Systematic Reviews; CRD42024523025). We conducted a systematic review and meta-analysis following the guidelines outlined in the Cochrane Handbook [18] and adhered to the 2020 PRISMA (Preferred Reporting Items for Systematic Reviews and Meta-Analyses) guidelines for systematic review reporting [19].

### Literature Search

We conducted a comprehensive systematic search across 5 databases: PubMed, Web of Science, CINAHL, Embase, and Cochrane. Our search included all forms of electronic communication, such as virtual reality, augmented reality, artificial intelligence, smartphone apps, TM, and SMS text messages. The search covered all records from the inception of each database to September 26, 2024. The complete search strategy for each database is detailed in Multimedia Appendices 1-5. Additionally, we manually searched the reference lists of retrieved articles.

### Inclusion and Exclusion Criteria

The inclusion criteria, defined using the PICOS (Patients, Implementation, Comparison, Outcomes, Study) format, were as follows: (1) *patients* diagnosed with type 1 diabetes; (2) *implementation* of the CC method via TM; (3) studies categorizing participants into experimental and control groups, including both usual and standard care (*comparison*); (4) HbA_1c_ levels (*outcomes*); and (5) RCTs (*study*).

The exclusion criteria were as follows: (1) studies that were incomplete, including research protocols and ongoing studies; (2) reports lacking sufficient details on patient outcome measures; and (3) studies with insufficient statistical data for quantitative outcomes, such as mean, SD, and median with range.

### Study Selection

First, the literature search results from all retrieved databases were exported into EndNote X9 (Clarivate Analytics) for management and duplicate removal. Two researchers (YL and YY) independently screened titles and abstracts, followed by a full-text review to confirm eligibility. A third researcher (FL) resolved any disagreements or discrepancies through deliberation until a consensus was reached.

### Data Extraction

Two researchers (YL and YY) independently extracted data from the 10 articles, and another researcher (FL) verified the extracted information. Discrepancies were resolved through discussion. If data were unavailable, the authors were contacted.

We extracted trial characteristics (study name, author, year, country, number of centers, design, duration, and sample size) and patient characteristics (age, sex, and duration of diabetes) from the selected studies. Additionally, we extracted details of the TM intervention (type, duration, and other specifics) and results (mean, SD, SE, 95% CIs, statistical significance at follow-up time points, primary and secondary outcomes, and validated measurement instruments).

### Outcomes

The primary outcome was the HbA_1c_ level. Secondary outcomes included treatment satisfaction (measured using a validated tool), total daily insulin dose (TDD), and time in range (TIR).

### Risk of Bias Assessment

Two reviewers independently assessed the risk of bias in RCTs using the revised Cochrane Risk of Bias (RoB) 2 tool and calculated the weighted Cohen κ coefficient to measure agreement [20]. In case of disagreement, a third reviewer (FL) facilitated discussions to reach a consensus. The RoB 2 tool evaluates 5 key areas of potential bias in RCTs: the randomization process, deviations from intended interventions, missing outcome data, outcome measurement, and selective reporting of outcomes. Each area includes a series of questions with 3 response options—“low,” “some concern,” and “high”—which classify the level of bias risk. Based on these classifications, studies are categorized as having “low,” “some concern,” or “high” overall risk of bias. The assessments in each area contribute to the overall judgment of bias risk in the study results. If no bias is identified in any area, the overall risk is deemed low. If at least one area raises some concern, the overall risk is categorized as “some concern.” If a high risk of bias is found in any area, the study is considered to have a high overall risk of bias [20].

We assessed publication bias using the Egger test [21] and examined contour-enhanced funnel plots [22]. This is particularly important in meta-analyses, as studies with positive and significant results are more likely to be published in high-impact journals compared with those with negative findings [23].

### Data Synthesis and Analysis

Statistical analysis was performed using the metafor package in R 4.4.1 software (R Foundation) [24]. For HbA_1c_ and TIR, the mean difference (MD) and 95% CI were calculated. Given that treatment satisfaction was measured using different validation scales, and TDD used other measurement methods and units, we calculated the standardized mean difference (SMD) and 95% CIs. When studies reported baseline and follow-up values but not change-from-baseline SDs, the missing SDs were calculated based on the baseline and follow-up SDs, and the average correlation coefficient (*r*) was estimated from other identified studies using the following formula:

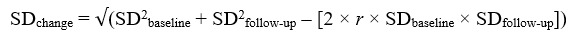

If the SE was reported instead of the SD, it was converted to SD using the following formula:

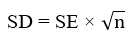

where n is the sample size. By contrast, if a 95% CI was reported instead of SD or SE, the SD can be calculated according to the Cochrane Manual [25]. Studies that did not report SDs, SEs, or 95% CIs were excluded from meta-analysis [25].

Given the expected heterogeneity in study populations and procedures, a random-effects model was used to combine the effect sizes and SDs of the studies. Heterogeneity was assessed by examining the forest plot and calculating the degree of inconsistency (*I*^2^) among studies. *I*^2^ represents the proportion of variability in study results attributable to heterogeneity rather than chance [26]. An *I*^2^ value of 0%-40% indicated low heterogeneity, 30%-60% indicated moderate heterogeneity, 50%-90% indicated substantial heterogeneity, and 75%-100% indicated considerable heterogeneity. To assess the influence of individual studies, we conducted a leave-one-out analysis, systematically excluding each study and reanalyzing the data set to determine whether any single study disproportionately affected the results. Sensitivity analyses were performed to evaluate the robustness of the findings. Additionally, subgroup analyses based on the type of intervention were conducted to explore potential sources of heterogeneity. Finally, meta-regression was used to examine the impact of demographic and intervention characteristics on variations in HbA_1c_ levels, aiming to identify underlying sources of heterogeneity.

## Results

### Study Selection

**Figure 1:**
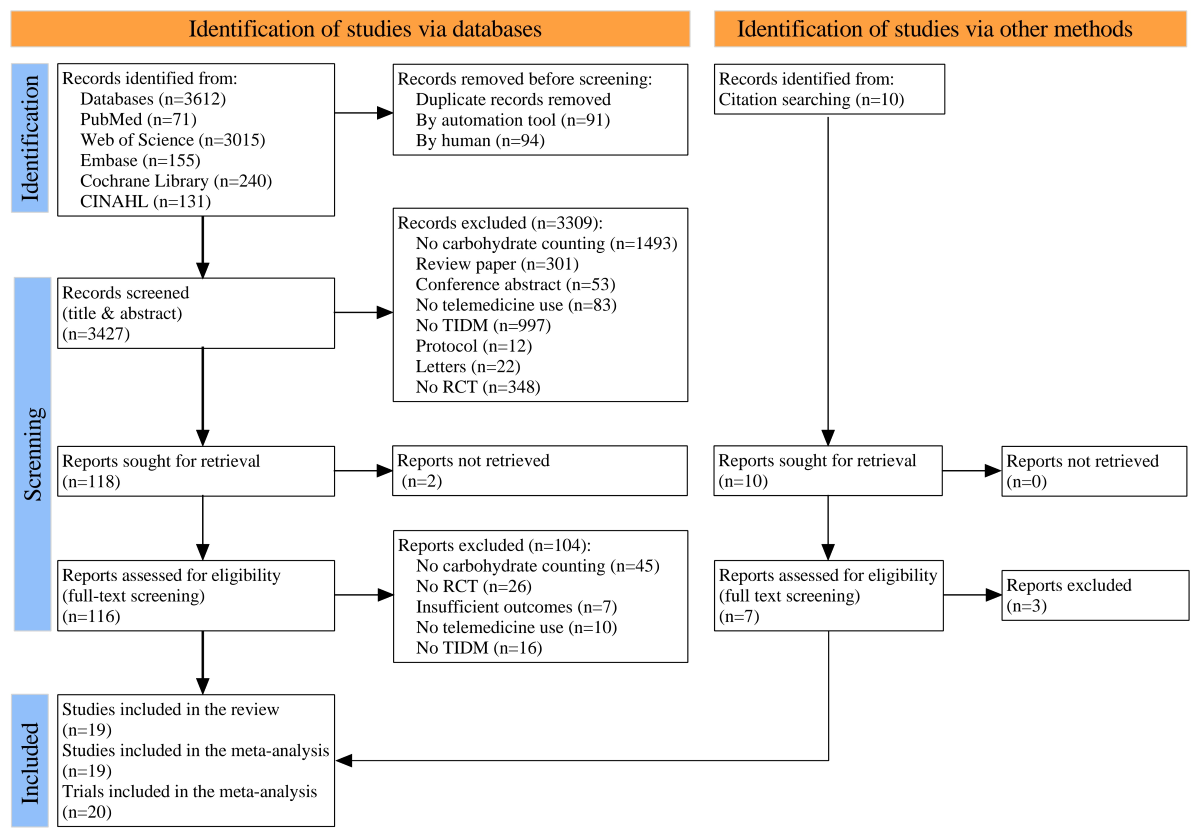
PRISMA (Preferred Reporting Items for Systematic Reviews and Meta-Analyses) flowchart illustrating the study selection process. RCT: randomized controlled trial; T1DM: type 1 diabetes mellitus.

The initial database search yielded 3612 records, with an additional 10 identified through reference list screening. After removing 185 duplicates, 3437 abstracts were screened, of which 3317 were excluded for not meeting the selection criteria, and 2 lacked full-text access. Subsequently, 118 full-text articles were assessed, with exclusions detailed in Figure 1. Ultimately, 19 articles met the inclusion criteria and were included in the final analysis. One of these articles reported 2 distinct intervention groups within a 3-arm trial, comparing both intervention groups against a control, resulting in the inclusion of 20 trials in the meta-analysis [27-45]. The interrater agreement between evaluators (YL and YY) was strong, with a κ value of 0.91.

### Characteristics of the Included Studies

Table 1 summarizes the characteristics of the 20 included studies, encompassing 1627 participants from 14 regions. The publication years ranged from 2004 to 2023, with over 50% (12/20, 60%) published after 2018. Geographically, the studies were conducted in Europe (n=12), America (n=6), and Asia (n=2). All studies were RCTs, with 17 applying a parallel-group design and 3 utilizing a crossover design. Regarding the study population, 7 focused on children and adolescents with T1DM, 11 on adults with T1DM, and 2 included participants of all ages with T1DM.

**Table 1:** Characteristics of the included studies.

Study	Country	Centers, n	RCT^a^ design	Population	Participants, n		Age (years), mean (SD)		Male, n (%)	HbA_1c_^b^, mean (SD)		Duration of diabetes (years), mean (SD)	
					Intervention group	Control group	Intervention group	Control group		Intervention group	Control group	Intervention group	Control group
Kowalska et al [27]	Poland	1	Parallel	Age <18 years and T1DM^c^ diagnosed at least 1 year prior	53	53	9.8 (4.3)	12.1 (3.7)	44 (41.5)	7.6 (1)	7.4 (1)	4.52 (2.7)	5.4 (3.5)
Alfonsi et al [28]	Canada	1	Crossover	Age 10-17 years and T1DM diagnosed at least 6 months prior	22	22	13.98 (1.57)	13.98 (1.76)	27 (61.4)	8.41 (1.8)	8.35 (1.32)	6.08 (4.14)	6.44 (4.45)
Charpentier et al [29]	France	17	Parallel	Age >18 years and T1DM diagnosed at least 1 year prior	59	61	32.26 (12.07)	36.8 (14.1)	43 (35.8)	9.11 (1.14)	8.91 (0.90)	14.7 (9.1)	16.9 (10.5)
Rossi et al [30]	England, Italy, and Spain	7	Parallel	Age ≥8 years with T1DM	67	63	35.4 (9.5)	36.1 (9.4)	56 (43.1)	8.2 (0.8)	8.4 (0.7)	17.1 (10)	15.8 (10.7)
de Oliveira et al [31]	Canada	1	Parallel	T1DM or LADA^d^ diagnosed at least 1 year prior	22	23	22.6^e^	38.7^e^	20 (44.4)	7.28	7.76	N/A^f^	N/A
Rossi et al [32]	Italy	12	Parallel	Age ≥18 years with T1DM	63	64	38.4 (10.3)	34.3 (10.0)	60 (47.2)	8.4 (0.1)	8.5 (0.1)	N/A	N/A
Gunawardena et al [33]	Sri Lanka	1	Parallel	Age 18-80 years and DM diagnosed at least 6 months prior	35	32	52 (12)	53 (11)	50 (74.6)	9.5 (1.6)	9.4 (1.3)	11 (6)	11 (7)
Lee et al [34]	Korea	1	Parallel	Age 19-79 years and T1DM diagnosed at least 1 year prior	18	18	45.4 (12.3)	43.1 (14.6)	17 (47.2)	9.2 (2.0)	8.8 (1.1)	16.0 (10.4)	18.2 (10.5)
Castensøe-Seidenfaden et al [35]	Denmark	6	Parallel	Age 14-22 years and T1DM diagnosed at least 1 year prior	76	75	17.6 (2.6)	17.6 (2.7)	70 (46.4)	8.3 (4.3)	7.7 (4.7)	81.1 (18.0)	76.2 (14.9)
Schmidt et al [36]	Denmark	2	Parallel	Age 18-65 years and T1DM diagnosed at least 1 year prior	22	8	42 (10)	46 (9)	14 (32.6)	8.8 (0.7)	9.1 (0.7)	21 (9)	14 (12)
Klee et al [37]	Switzerland	1	Crossover	Age 10-18 years and T1DM diagnosed at least 6 months prior	16	16	13.3 (2.3)	13.3 (2.3)	N/A	8.8 (0.7)	8.8 (0.7)	N/A	N/A
Hommel et al [38]	Denmark	1	Parallel	Age ≥18 years and T1DM diagnosed at least 1 year prior	84	84	46.9 (14.4)	47.1 (12.7)	96 (57.1)	8.9 (0.7)	9.0 (0.8)	23.4 (13.9)	22.0 (13.9)
Boukhors et al [39]	Canada	1	Crossover	T1DM diagnosed at least 1 year prior	10	10	39.3 (10.1)	39.3 (10.1)	14 (70.0)	7.7 (0.9)	7.7 (0.9)	N/A	N/A
Montanari et al [40]	Brazil	1	Parallel	Age 18-45 years with T1DM	33	35	26.0 (7.04)	27.82 (5.98)	40 (58.8)	9.6 (1.5)	9.0 (0.5)	16.86 (6.07)	18.41 (6.54)
Montanari et al [40]	Brazil	1	Parallel	Age 18-45 years with T1DM	43	35	26.81 (7.06)	27.82 (5.98)	50 (64.1)	9.0 (0.5)	9.0 (0.5)	16.47 (7.55)	18.41 (6.54)
Secher et al [41]	Denmark	5	Parallel	Age ≥18 years and T1DM diagnosed at least 1 year prior	41	42	47.2 (15.1)	44.6 (13.5)	55 (26.4)	8.0 (0.74)	8.2 (0.52)	16 (12.59)	16.5 (13.3)
Chatzakis et al [42]	Greece	1	Parallel	Age 7-17 years with T1DM	40	40	13.8 (3)	13.2 (2.7)	39 (48.8)	8.25 (0.8)	7.9 (0.62)	6.7 (4.4)	6.1 (3.8)
Wadwa et al [43]	America	3	Parallel	Age 2-6 years and T1DM diagnosed at least 1 month prior	68	34	3.84 (1.23)	4.06 (1.25)	52 (51.0)	7.5 (1.2)	7.7 (0.9)	N/A	N/A
Ballesta et al [44]	Spain	1	Parallel	Age >18 years and T1DM diagnosed at least 6 months prior	26	29	52.5 (12.4)	50.1 (12.5)	27 (49.1)	7.52 (0.72)	7.61 (0.69)	24.5 (12.2)	20.0 (10.5)
Enander et al [45]	Sweden	3	Parallel	Age ≤18 years with T1DM	14	14	14.1 (3.2)	13.2 (4.0)	N/A	7.2 (0.6)	7.7 (1.0)	N/A	N/A

^a^RCT: randomized controlled trial.

^b^HbA_1c_: hemoglobin A_1c_.

^c^T1DM: type 1 diabetes mellitus.

^d^LADA: latent autoimmune diabetes in adults.

^e^SD was not reported in the study.

^f^N/A: not applicable.

### Intervention Characteristics

CC interventions using different TM models exhibited considerable variability in glycemic control outcomes among patients with T1DM (Table 2). The interventions were categorized into 3 groups based on their content: smartphone apps (n=13), web-based systems (n=3), and connected or wearable glucometers (n=4).

Currently, smartphone apps are the most widely used, encompassing various types. These include the iSpy mobile app, OneTouch Reveal, Young with Diabetes mHealth app, Webdia mHealth app, GLIC APP, MySugr, Euglia, Social Diabetes, and the “artificial pancreas.” The web-based category comprises integrated network systems such as the ELKa system, cloud-based platforms, and computer programs accessible via the internet. Internet-connected glucometers, such as the Accu-Chek Connect by Roche, integrate self-monitoring of BG results with additional features, including an insulin calculator and a food diary. These tools assist users in calculating carbohydrate intake and managing BG levels more effectively while enabling clinicians to review accurate BG patterns for treatment adjustments [46].

**Table 2:** Characteristics of carbohydrate-counting interventions using telemedicine for type 1 diabetes mellitus.

Study	Technology	Control	Length	Primary outcome	Secondary outcomes		
					Satisfaction treatment	Time in range	Total daily insulin dose
Kowalska et al [27]	ELKa system	Usual care	26 weeks	HbA_1c_^a^	N/A^b^	N/A	✓
Alfonsi et al [28]	iSpy mobile app	Usual care	3 months	HbA_1c_	N/A	N/A	N/A
Charpentier et al [29]	Diabeo software	Paper diaries followed up at the hospital outpatient clinic	6 months	HbA_1c_	N/A	N/A	N/A
Rossi et al [30]	Diabetes Interactive Diary TM^c^ system	Usual care	6 months	HbA_1c_	✓	N/A	N/A
de Oliveira et al [31]	OneTouch Reveal mobile phone app	Usual care	12 months	HbA_1c_	N/A	N/A	✓
Rossi et al [32]	Diabetes Interactive Diary TM system	Usual care	6 months	HbA_1c_	✓	N/A	N/A
Gunawardena et al [33]	A smart glucose manager–based mobile app	Usual care	6 months	HbA_1c_	N/A	N/A	N/A
Lee et al [34]	Cloud system	Usual care	12 weeks	HbA_1c_	✓	✓	✓
Castensøe-Seidenfaden et al [35]	Young with Diabetes mHealth app	Usual care	12 months	HbA_1c_	N/A	N/A	N/A
Schmidt et al [36]	Accu-Chek Aviva Expert bolus calculator device	Hours of structured small-group instructions	16 weeks	HbA_1c_	✓	N/A	✓
Klee et al [37]	Webdia mHealth app	Usual care	3 months	HbA_1c_	N/A	N/A	N/A
Hommel et al [38]	StenoABC automated bolus calculator	Attending a 3.5-hour training session	12 months	HbA_1c_	N/A	N/A	N/A
Boukhors et al [39]	The computer program was accessible via the internet	Patients will record their blood glucose in their journals and adjust it according to the algorithm	4 months	HbA_1c_	N/A	N/A	✓
Montanari et al [40]	Intelligent glucometer (COMBO)	Usual care	6 months	HbA_1c_	N/A	✓	N/A
Montanari et al [40]	GLIC APP	Usual care	6 months	HbA_1c_	N/A	N/A	N/A
Secher et al [41]	MySugr app	Routine care	26 weeks	HbA_1c_	N/A	✓	✓
Chatzakis et al [42]	Euglia app	Usual care	12 months	HbA_1c_	✓	N/A	N/A
Wadwa et al [43]	AP app	Usual care	16 weeks	HbA_1c_	N/A	✓	N/A
Ballesta et al [44]	Social Diabetes app	Usual care	6 months	HbA_1c_	N/A	N/A	N/A
Enander et al [45]	Cozmo pump	Usual care	12 months	HbA_1c_	N/A	N/A	✓

^a^HbA_1c_: hemoglobin A_1c_**_._**

^b^N/A: not applicable.

^c^TM: telemedicine.

### Risk of Bias

**Figure 2:**
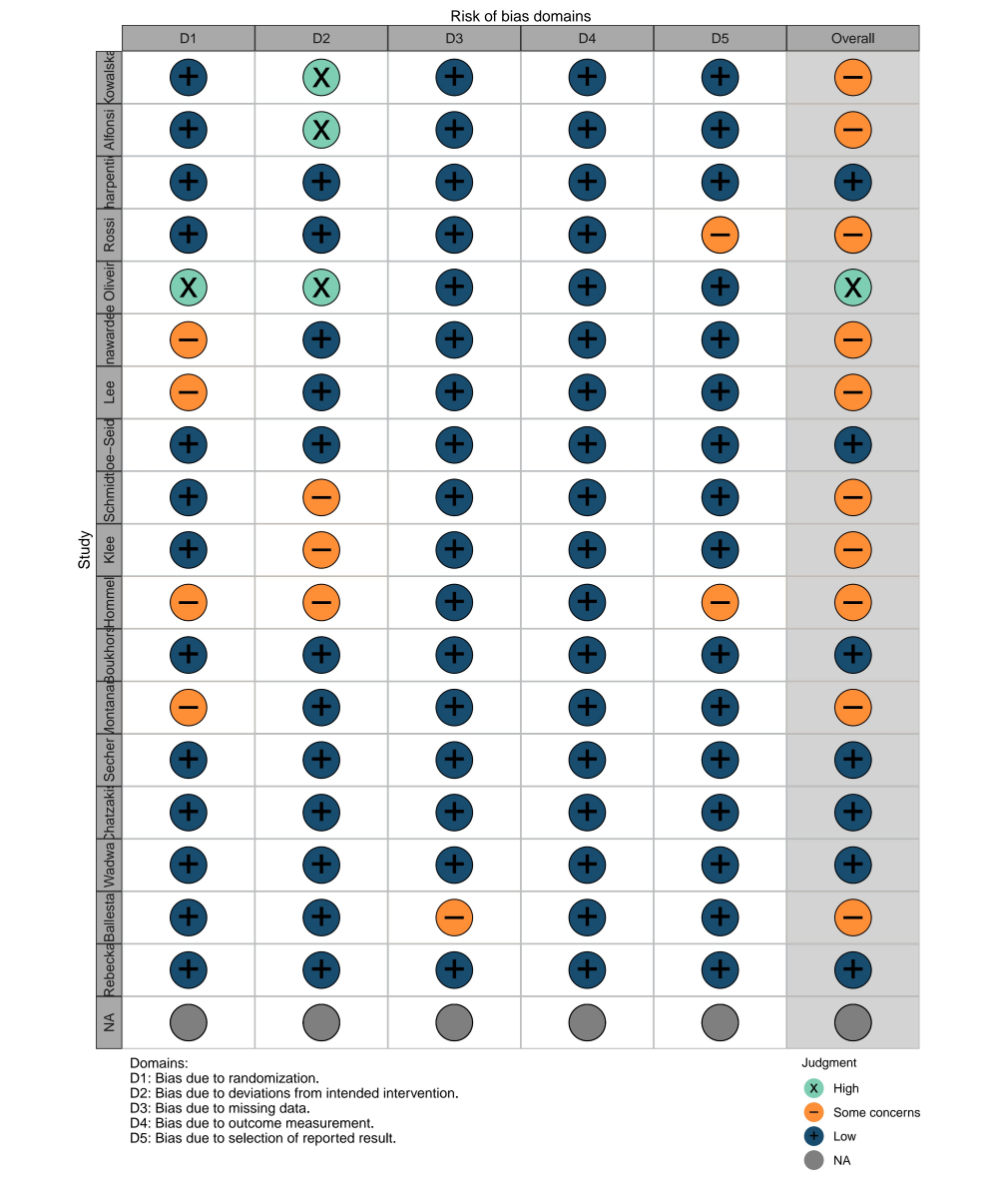
Risk of bias graph.

**Figure 3:**
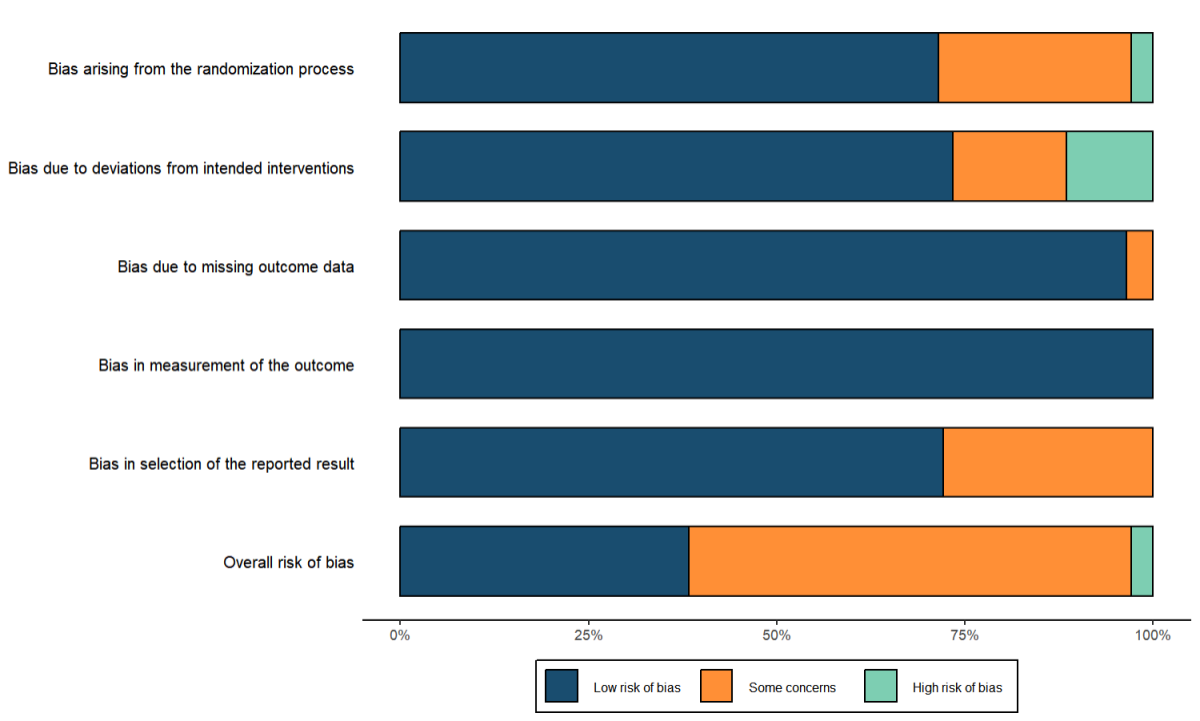
Risk of bias summary.

Figure 2 presents the risk of biased outcomes for the 19 included articles, highlighting variability in study quality. Of these, 7 articles (37%) exhibited a low risk of bias, 11 (58%) demonstrated some risk of bias, and 1 (5%) had a high risk of bias. The high risk of bias was primarily attributed to inadequate descriptions of randomization methods, the absence of participant and researcher blinding during intervention allocation, and deviations from the planned intervention. Given the impracticality of blinding participants in TM interventions, all trials were conducted using an open-label design, resulting in at least one identified risk of bias per study. Our assessment revealed that 15 out of 19 articles (79%) adequately reported and described appropriate randomization methods, while 13 (68%) effectively communicated assignment concealment procedures. Additionally, 11 studies (58%) adhered to the intention-to-treat principle. As shown in Table 3, interrater agreement for risk of bias assessments was high, with Cohen κ values ranging from 0.642 to 1.00 across domains. A risk of bias summary is presented in Figure 3.

**Table 3:** Risk of bias of randomized controlled trials.

Study	D1: Randomization process	D2: Deviations from intended interventions	D3: Missing outcome data	D4: Measurement of the outcome	D5: Selection of the reported result	D6: Overall bias
Kowalska et al [27]	Low	High	Low	Low	Low	Some concerns
Alfonsi et al [28]	Low	High	Low	Low	Low	Some concerns
Charpentier et al [29]	Low	Low	Low	Low	Low	Low
Rossi et al [30]	Low	Low	Low	Low	Some concerns	Some concerns
de Oliveira et al [31]	High	High	Low	Low	Low	High
Rossi et al [32]	Low	Low	Low	Low	Some concerns	Some concerns
Gunawardena et al [33]	Some concerns	Low	Low	Low	Low	Some concerns
Lee et al [34]	Some concerns	Low	Low	Low	Low	Some concerns
Castensøe-Seidenfaden et al [35]	Low	Low	Low	Low	Low	Low
Schmidt et al [36]	Low	Some concerns	Low	Low	Low	Some concerns
Klee et al [37]	Low	Some concerns	Low	Low	Low	Some concerns
Hommel et al [38]	Some concerns	Some concerns	Low	Low	Some concerns	Some concerns
Boukhors et al [39]	Low	Low	Low	Low	Low	Low
Montanari et al [40]	Some concerns	Low	Low	Low	Low	Some concerns
Secher et al [41]	Low	Low	Low	Low	Low	Low
Chatzakis et al [42]	Low	Low	Low	Low	Low	Low
Wadwa et al [43]	Low	Low	Low	Low	Low	Low
Ballesta et al [44]	Low	Low	Some concerns	Low	Low	Some concerns
Enander et al [45]	Low	Low	Low	Low	Low	Low
κ	0.747	1.00	0.642	1.00	0.826	0.912

**Figure 4:**
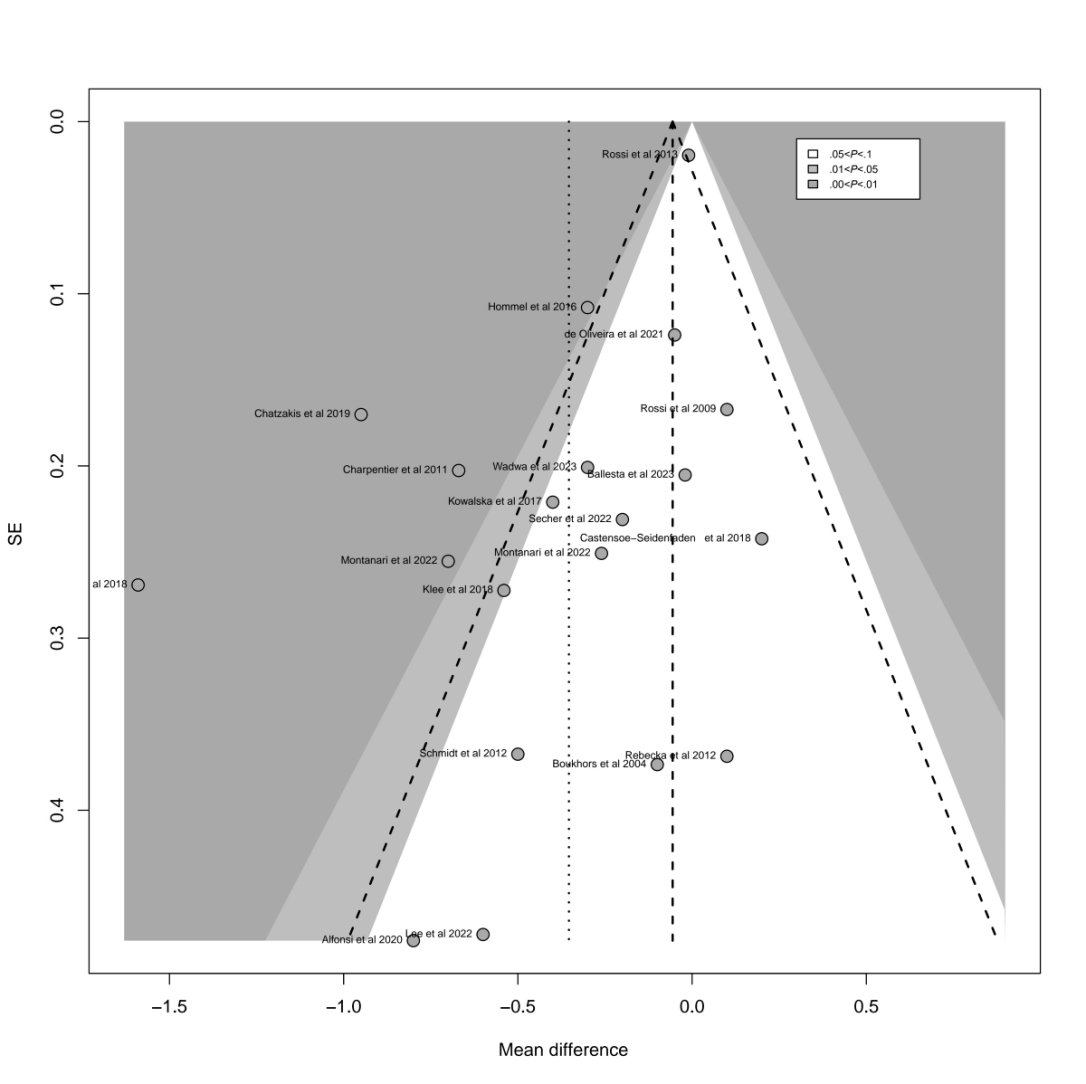
Funnel plot for the detection of publication bias for hemoglobin A_1c_ (HbA_1c_).

**Figure 5:**
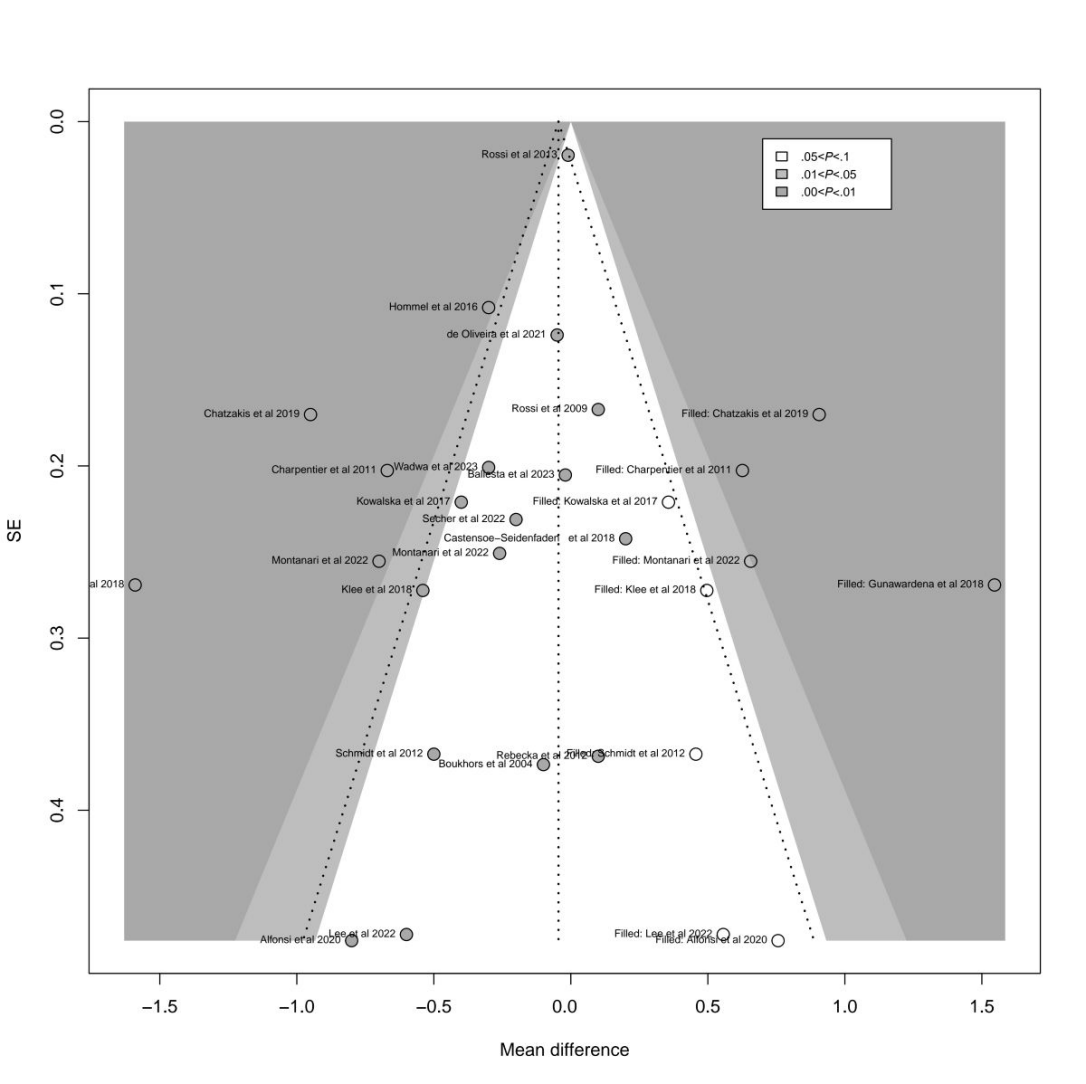
Contour-enhanced funnel plot for detecting publication bias in hemoglobin A_1c_ (HbA_1c_) studies.

Egger test revealed significant asymmetry in study distribution (*P*=.003). This asymmetry was also evident in the contour-enhanced funnel plot of HbA_1c_ (Figure 4; see also [27-45]), suggesting a degree of publication bias consistent with the Egger test results. In the profile-enhanced funnel plot after applying the trim-and-fill method (Figure 5; see also [27-45]), 9 additional studies (represented by modest circles) were required to correct the observed asymmetry. While some studies fell within the nonsignificant region (white areas), indicating that no significant studies were left unpublished, others appeared in the statistically significant region (gray area), suggesting that certain significant studies may not have been published. This implies that factors beyond publication bias may have contributed to the observed funnel plot asymmetry for HbA_1c_.

### Primary Outcome: HbA_1c_

#### Overview

**Figure 6:**
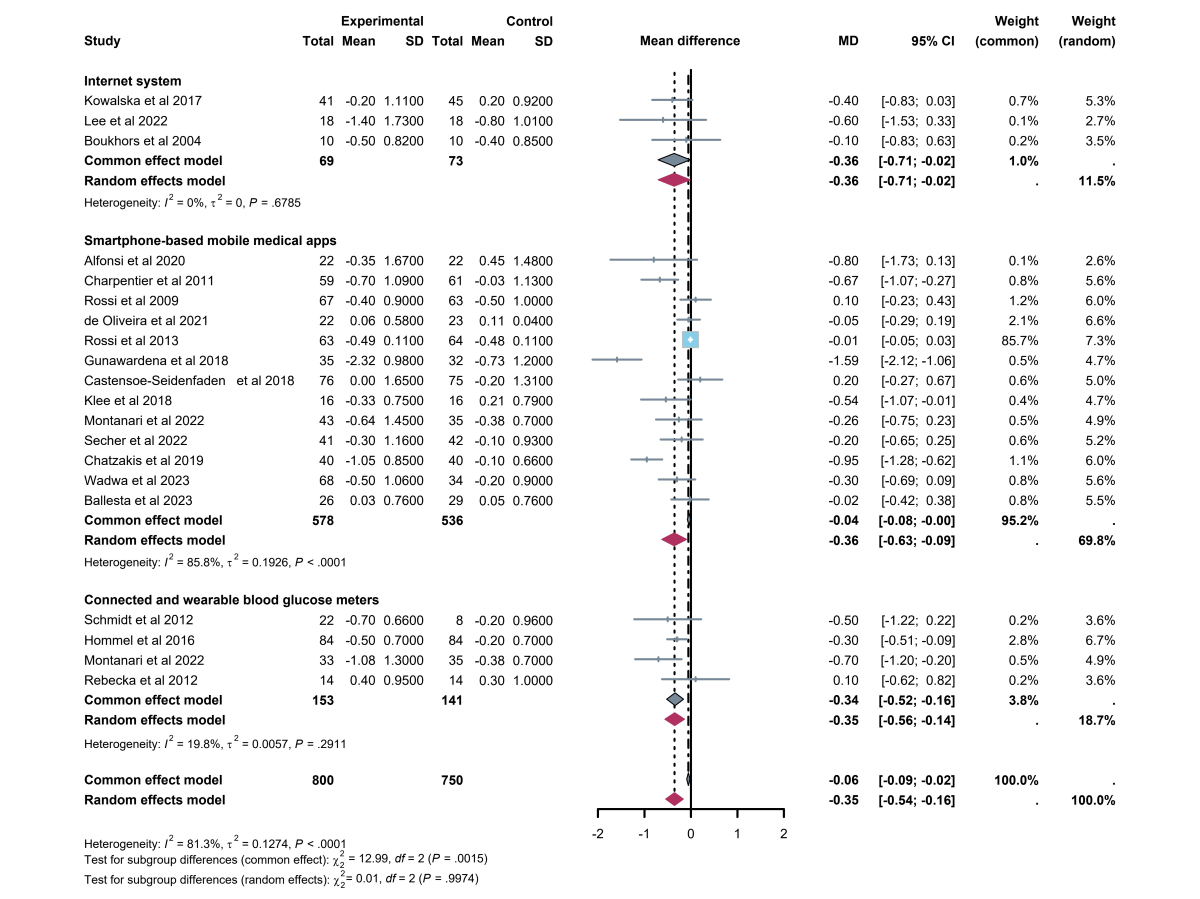
Differences in mean hemoglobin A_1c_ (HbA_1c_) levels between the telemedicine intervention group and the control group.

A meta-analysis of 20 trials assessing HbA_1c_ levels found that CC intervention through TM led to an overall reduction of –0.35% (95% CI –0.54% to –0.16%) compared with the control group (Figure 6; see also [27-45]). However, the heterogeneity test indicated substantial variability among studies (*I*^2^=81%, *P*<.01).

#### Subgroup Analyses

Considerable heterogeneity was observed among the studies (*I*^2^=81%, *P*<.001). Among intervention types, smartphone apps showed the most substantial reduction in HbA_1c_ (–0.36%, 95%CI –0.63% to –0.09%), with a significant effect; however, heterogeneity remained high (*I*^2^=86%, *P*<.001). Interventions using connected and wearable glucose meters also significantly reduced HbA_1c_ (–0.34%, 95% CI –0.52% to –0.16%), but with low heterogeneity (*I*^2^=20%, *P*=.29). The smallest reduction in HbA_1c_ was observed with network-based systems (–0.36%, 95% CI –0.71% to –0.02%), which also had a significant effect, with very low heterogeneity (*I*^2^=0%, *P*=.68). Despite these differences, the subgroup analysis of intervention approaches did not indicate significant heterogeneity (*P*>.99; Figure 6).

#### Sensitivity Analyses

**Figure 7:**
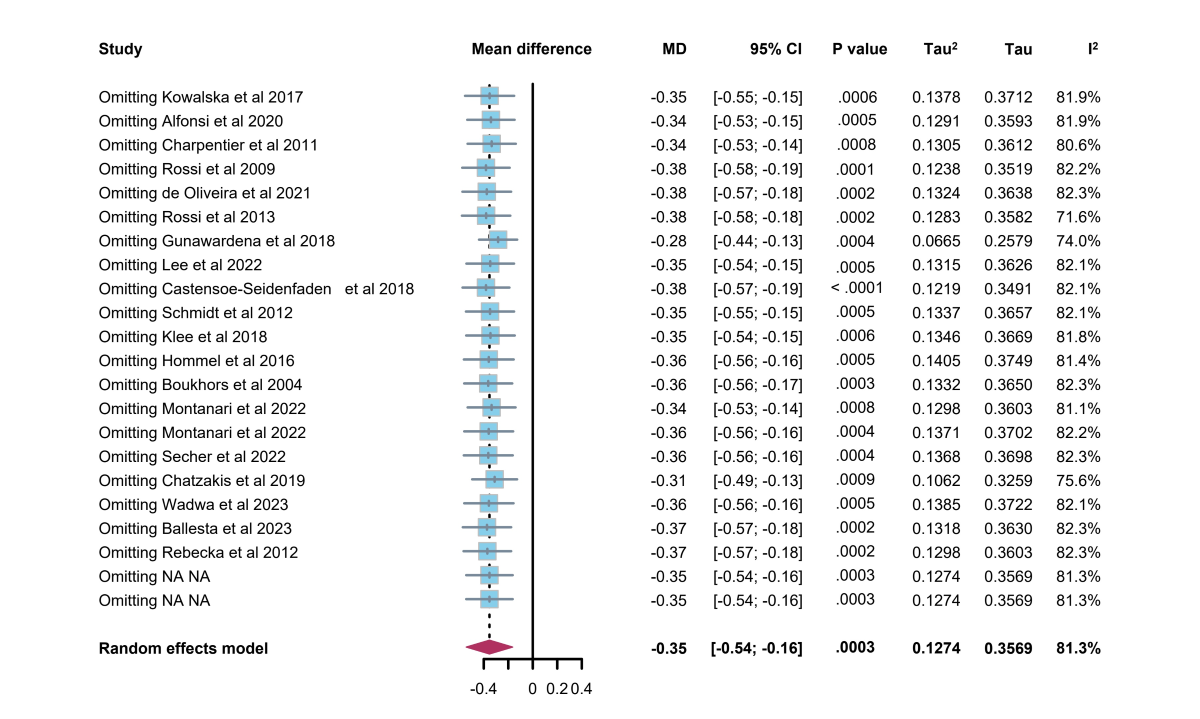
Sensitivity analysis assessing the influence of individual studies by removing them one at a time from the summary estimates (mean difference [MD] and 95% CI) of hemoglobin A_1c_ (HbA_1c_).

Figure 7 (see also [27-45]) presents the results of the sensitivity analyses, performed by sequentially removing 1 study (trial) at a time. The random-effects model demonstrated that the MD in HbA_1c_ levels remained stable throughout, indicating that the findings were robust, reliable, and not disproportionately influenced by any single study.

#### Meta-Analysis

We conducted a univariate meta-regression analysis to explore the influence of population and intervention characteristics on heterogeneity (Table 4). The results indicated that trial region was a significant factor affecting heterogeneity (*P*<.05). However, follow-up duration (*P*=.40), sample size (*P*=.34), percentage of male participants, baseline HbA_1c_ level, intervention type, age, and risk of bias did not significantly contribute to heterogeneity. While trials conducted in Europe (n=12) and America (n=6) showed comparable MDs (–0.31% and –0.26%, respectively), trials from Asia (n=2) demonstrated a significantly greater reduction in HbA_1c_ with TM intervention (MD –1.26%, 95% CI –1.88% to –0.64%).

**Table 4:** Meta-regression results and pooled estimates in study subgroups for HbA_1c_^a^.

Variable		Sample, n	Mean difference (95% CI), %		*P* value		*I*^2^ statistic, %
Duration of follow-up (months)		20	0.03 (–0.03 to 0.09)		.40		82.51
Sample size		20	0.00 (0.00 to 0.00)		.34		82.69
**Risk of bias**		20					82.98
	Low	7	–0.32 (–0.66 to 0.02)	.07			
	Some concerns	12	–0.41 (–0.67 to –0.15)	<.001			
	High	1	–0.05 (–0.84 to 0.74)	.90			
**Baseline HbA_1c_(%)**		20					81.79
	>8.0	14	–0.44 (–0.67 to –0.22)	<.001			
	<8.0	6	–0.14 (–0.48 to 0.20)	.42			
**Age (years)**		20					82.60
	>18	11	–0.39 (–0.65 to –0.13)	<.001			
	<18	7	–0.39 (–0.73 to –0.05)	.03			
	All age stages	2	–0.07 (–0.69, 0.55)	.83			
**Location**		20					74.90
	America	6	–0.31 (–0.62 to 0.00)	.04			
	Europe	12	–0.26 (–0.46 to –0.06)	.01			
	Asia	2	–1.26 (–1.88 to –0.64)	<.001			
**TM^b^ type**		20					84.22
	Internet system	3	–0.36 (–0.94 to 0.23)	.23			
	Smartphone-based mobile medical apps	13	–0.35 (–0.59 to –0.11)	<.001			
	Connected and wearable blood glucose meters	4	–0.37 (–0.83 to 0.10)	.12			

^a^HbA_1c_: hemoglobin A_1c_.

^b^TM: telemedicine.

### Secondary Outcomes

#### Satisfaction With Diabetes Treatment

**Figure 8:**
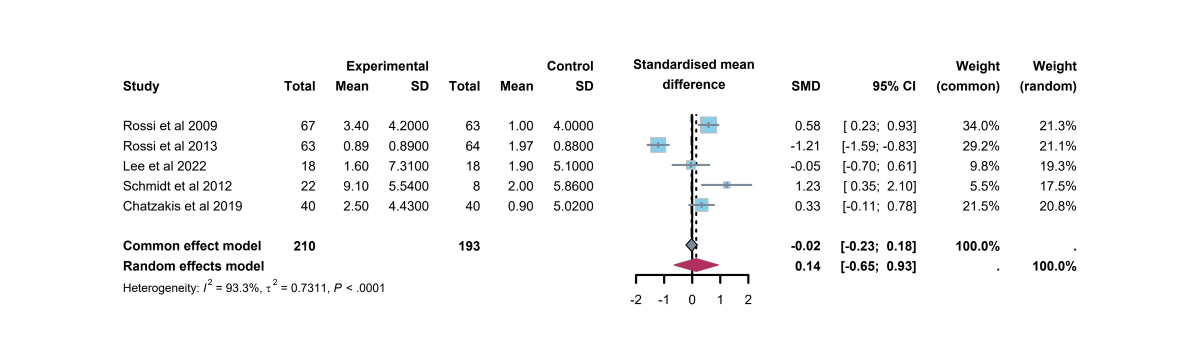
Differences in mean satisfaction with diabetes treatment between the telemedicine intervention group and the control group. SMD: standardized mean difference.

Five trials [30,32,34,36,38] examined the effect of TM-based CC interventions on satisfaction with diabetes treatment. The Diabetes Treatment Satisfaction Questionnaire (DTSQ), a widely used 8-item scale, was the primary assessment tool. Of these items, 6 contributed to a total score ranging from 0 (indicating high dissatisfaction) to 36 (indicating high satisfaction), while the remaining 2 were analyzed separately to assess the perceived frequency of hyperglycemia and hypoglycemia episodes [47]. Both the DTSQs (state version) and DTSQc (change version) were evaluated. The findings revealed a modest increase in patient satisfaction with TM-based CC interventions, with a change in satisfaction score of 0.14 (95% CI –0.65 to –0.93). However, this difference was not statistically significant compared with standard care. While all 5 studies reported improved treatment satisfaction, only 3 [30,36,42] demonstrated a significant difference (Figure 8).

#### Time in Range

**Figure 9:**
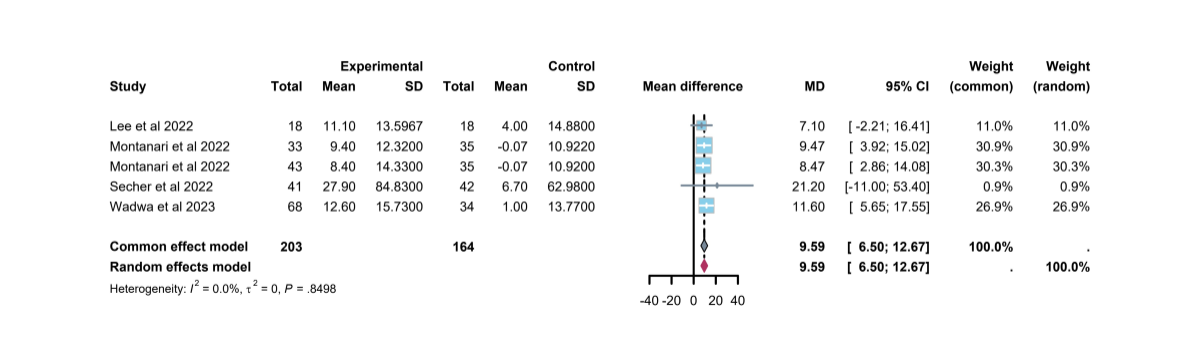
Differences in mean time in range between the telemedicine intervention group and the control group. MD: mean difference.

Five trials [34,40,41,43] assessed the effect of TM-based CC interventions on glucose TIR events. TIR represents the percentage of time that BG remains within the target range (70-180 mg/dL in all 5 trials) over 24 hours, providing a visual depiction of daily glucose fluctuations in individuals with T1DM. The 2019 International Congress on Advanced Diabetes Technology and Treatment (ATTD) and the 2020 China Guidelines for the Prevention and Treatment of Type 2 Diabetes Mellitus (T2DM) [48] strongly recommend integrating TIR with self-monitoring of blood glucose, continuous glucose monitoring, and HbA_1c_ to comprehensively assess glycemic control. This combination has become a key reference index for clinical diabetes management. According to the International Consensus on Continuous Glucose Monitoring, a 5% increase in TIR is associated with a reduced risk of chronic diabetic complications [49]. The findings demonstrated a significant increase in TIR, with a mean improvement of 9.59% (95% CI 6.50% to 12.67%). Moreover, TM-based CC interventions significantly enhanced TIR and reduced the incidence of diabetic complications compared with conventional care. Four trials (3 studies [34,40,43]) reported statistically significant improvements in TIR, whereas 1 trial [41] did not observe significant changes (Figure 9).

#### Total Daily Insulin Dose

**Figure 10:**
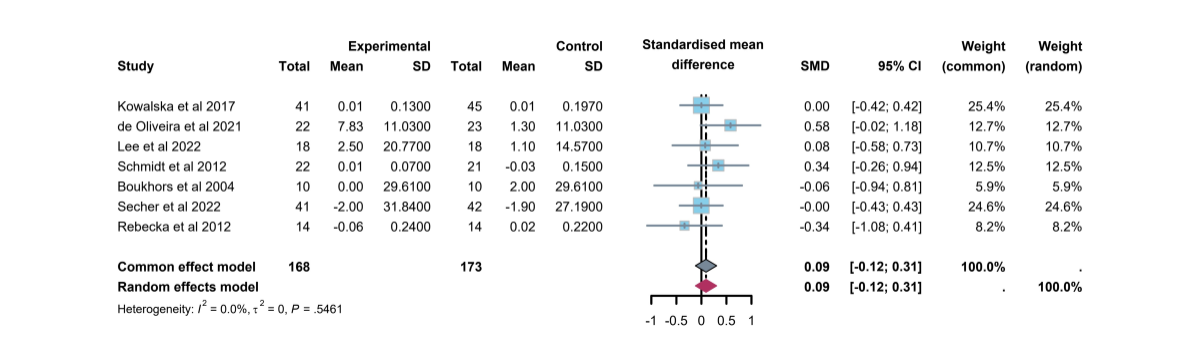
Differences in mean total daily insulin dose between the telemedicine intervention and control care groups.

Seven trials [27,31,34,36,39,41,45] were included in the meta-analysis of TDD. All studies reported insulin doses either as units per day or as units per kilogram per day. The results indicated a slight increase in TDD (0.09, 95% CI −0.12 to 0.31) with the TM-delivered CC intervention. However, no significant difference was observed between the TM-based CC intervention and usual care. While 4 studies [27,36,39,41] reported no significant changes in TDD, 3 studies [31,34,45] showed an increase, though none reached statistical significance (Figure 10).

### Adverse Effects

Three studies [28,33,39] did not mention hypoglycemia, and inconsistencies in its definition and reporting prevented a meta-analysis. Eight studies [27,32,34,40-44] defined hypoglycemia based on objective BG values, with cut-off thresholds ranging from <3.9 mmol/L to <2.5 mmol/L. Meanwhile, 7 studies [29-31,35-38] defined hypoglycemic events as those requiring assistance from another person.

## Discussion

### Summary and Interpretation of Findings

This meta-analysis of RCTs evaluating TM-based CC interventions for BG control in patients with T1DM included data from 20 studies completed on September 26, 2024. TM-based CC interventions significantly reduced HbA_1c_ levels by –0.35% (95% CI –0.54% to –0.16%) and improved glycemic control compared with controls. Considerable heterogeneity was observed among trials (*I*^2^=81%, *P*<.01), which may have led to an underestimation of the intervention effect and contributed to the asymmetry in the funnel plot, potentially due to factors other than publication bias. TM-based CC interventions significantly improved TIR (9.59%, 95% CI 6.50%-12.67%). However, no significant differences were found in hypoglycemia, treatment satisfaction, or TDD, possibly due to the short duration of the included trials.

The included studies were categorized based on the type of TM intervention: smartphone apps, connected and wearable glucometers, and web-based systems. Subgroup analysis showed that all intervention types had statistically significant effects on HbA_1c_ levels. Meta-regression analysis indicated that the study region was significantly associated with changes in HbA_1c_ levels. In terms of average HbA_1c_ reduction, Asian participants appeared to benefit more than North American and European populations. However, this conclusion should be interpreted with caution, as only 2 studies from Asia were included, compared with 12 from Europe and 6 from North America.

### Comparison With Prior Work

#### HbA_1c_

The findings of this study suggest that remote medical interventions for CC can significantly reduce HbA_1c_ levels, aligning with previous research [50,51]. However, the magnitude of reduction varies across studies. For instance, Eberle and Stichling [52] reported a significant HbA_1c_ reduction in patients with T1DM and T2DM (MD –0.64%, 95% CI –1.01% to –0.26%), whereas Lee et al [53] observed a smaller effect in patients with T1DM (MD –0.18%, 95% CI –0.33% to –0.04%). These discrepancies may be attributed to differences in patient characteristics, intervention implementation, and study sample sizes. Additionally, subgroup analysis in this study indicated that smartphone app–based remote interventions significantly reduced HbA_1c_ levels in patients with T1DM, a finding that contrasts with Hou et al [54], who reported minimal differences between intervention and control groups. This inconsistency may stem from the inclusion of fewer and lower-quality studies, contributing to high heterogeneity.

#### Satisfaction With Diabetes Treatment

The results indicated that CC interventions delivered via TM did not significantly improve treatment satisfaction in patients with T1DM, consistent with findings from other studies. Similarly, a recent study by Zhang et al [55] on the effects of TM in children and adolescents with T1DM also reported no significant improvement in treatment satisfaction.

#### Time in Range

Our findings indicate that remote medical intervention for CC significantly improved TIR, aligning with previous research. A recent study on the impact of a closed-loop insulin system in patients with T1DM reported similar effects (MD 10.32%, 95% CI 8.70%-11.95%) [56]. This suggests that remote medical intervention can help patients better manage BG fluctuations and increase the time their blood sugar remains within target ranges.

#### Total Daily Insulin Dose

This study found that CC interventions using TM had no statistically significant effect on TDD in patients with T1DM. However, limited research has examined TDD as an outcome measure.

### Strengths and Limitations

This study has several strengths. First, our research strategy involved an extensive search across multiple databases, enhancing the comprehensiveness of the review. Second, we adhered to rigorous systematic review and meta-analysis standards following PRISMA (Multimedia Appendix 6) and Cochrane guidelines. Third, we conducted comprehensive sensitivity analyses to ensure the robustness of our findings. Finally, subgroup and meta-regression analyses were performed to validate our results.

This study has several limitations. The primary limitation is the considerable heterogeneity among the included studies. Despite conducting subgroup analyses and meta-regression to explore potential sources of heterogeneity, the results should be interpreted with caution due to the influence of uncontrolled or unmeasured factors. Additionally, our review excluded RCT registries, ongoing studies, and gray literature. While the inclusion of gray literature in systematic reviews remains debated [57], limiting the analysis to published studies may introduce publication bias. Furthermore, we restricted our search to English-language publications, which may affect the generalizability of our findings. Another limitation is the potential subjectivity in bias assessments using the RoB2 tool. Finally, many trials had small sample sizes, short durations, and lacked blinding. Most studies were conducted in developed countries, reflecting the limited availability and feasibility of technology-based interventions in low-income settings.

### Implications for Practice and Future Research

Our findings have several practical implications. First, remote medical interventions for CC demonstrate beneficial effects on glycemic control in patients with T1DM. Thus, we recommend incorporating TM-based CC interventions into long-term glucose management strategies. However, successful implementation requires consideration of factors such as patient learning ability, adaptability, acceptance of technology, and the level of medical team support. As the time needed to achieve independent glucose management varies among individuals, long-term studies are warranted to further assess these interventions [55].

Recent cross-sectional research on T1DM indicates that the likelihood of diabetic retinopathy increases with rising HbA_1c_ levels. The United Kingdom Prospective Diabetes Study (UKPDS) [58] found that a 1% reduction in average HbA_1c_ was associated with a 21% reduction in diabetes-related deaths, a 14% reduction in the risk of myocardial infarction, and a 37% reduction in microvascular complications in patients with T2DM. If remote medical care–based CC interventions were implemented universally for patients with T1DM, they could potentially reduce diabetes-related deaths by 7.4%, myocardial infarction risk by 4.9%, and microvascular complications by 13%. Such interventions may also improve glycemic control, lower the risk of macrovascular and microvascular complications, and enhance quality of life. However, other important outcomes, including hypoglycemia incidence, TIR, quality of life, and self-management behaviors, remain understudied and require further evaluation. Additional research should also focus on the needs and characteristics of special populations, such as children and older adults, to enhance acceptance and effectiveness [46].

Given these findings, our results suggest that remote medical care–based CC interventions hold significant potential for clinical application. Future efforts should focus on advancing technological innovation to develop more intelligent and user-friendly remote medical devices and apps, such as integrating artificial intelligence to provide personalized treatment recommendations. Additionally, strengthening international collaboration is essential to facilitate global implementation, particularly in low-income countries and remote areas. Encouraging patients to integrate these interventions into their daily lives and work could further enhance glycemic management in T1DM. Future research should prioritize evaluating the long-term effects and cost-effectiveness of remote medical interventions in diabetes management. Moreover, exploring their combined use with other diabetes management strategies could provide further insights. Large-scale, multicenter, and long-term follow-up clinical trials are necessary to assess their efficacy and safety in glycemic control for patients with T1DM. These findings would offer critical evidence to support policy makers in promoting and expanding the use of remote medical care in diabetes management.

### Conclusions

Our systematic review and meta-analysis suggest that TM-based CC interventions can be effective for glycemic control in patients with T1DM in RCTs. However, a robust, large-scale trial is needed to draw definitive conclusions. These findings may inform the development of new strategies to enhance T1DM management.

## Appendix

Multimedia Appendix 1 PubMed search trail (search updated 26/09/2024).

Multimedia Appendix 2 Web of Science Core Collection search trail (search updated 26/09/2024).

Multimedia Appendix 3 Embase search trail (search updated 26/09/2024).

Multimedia Appendix 4 CINAHL search trail (search updated 26/09/2024).

Multimedia Appendix 5 Cochrane Library search trail (search updated 26/09/2024).

Multimedia Appendix 6 PRISMA (Preferred Reporting Items for Systematic Reviews and Meta-Analyses) checklist.

## Abbreviations

ADA: American Diabetes Association
ATTD: Advanced Diabetes Technology and Treatment
BG: blood glucose
CC: carbohydrate counting
DTSQ: Diabetes Treatment Satisfaction Questionnaire
HbA_1c_: hemoglobin A_1c_
MD: mean difference
PICOS: Patients, Implementation, Comparison, Outcomes, Study
PRISMA: Preferred Reporting Items for Systematic Reviews and Meta-Analyses
PROSPERO: International Prospective Register of Systematic Reviews
RCT: randomized controlled trial
SMD: standardized mean difference
T1DM: type 1 diabetes mellitus
T2DM: type 2 diabetes mellitus
TDD: total daily insulin dose
TIR: time in range
TM: telemedicine
UKPDS: United Kingdom Prospective Diabetes Study
