# Supplementary material for: Effectiveness of Telemedicine-Delivered Carbohydrate-Counting Interventions in Patients With Type 1 Diabetes: Systematic Review and Meta-Analysis
Source: J Med Internet Res. 2025 Apr 10;27:e59579. doi: 10.2196/59579 (PMC12022529; doi:10.2196/59579)
Supplement: Multimedia Appendix 1 [file jmir_v27i1e59579_app1.docx]

Table 1. PubMed Search Trail (Search updated 26/09/2024)

| Search # | MeSH Terms and Key Words | Articles Revealed |
| --- | --- | --- |
| #1 | ((((((((Diabetes Mellitus[MeSH Terms]) OR (Diabetes Mellitus, Type 1[MeSH Terms])) OR (Diet, Diabetic[Title/Abstract])) OR (Latent Autoimmune Diabetes in Adults[Title/Abstract])) OR (Diabetes Mellitus, Insulin-Dependent[Title/Abstract])) OR (Type 1 Diabetes Mellitus[Title/Abstract])) OR (Diabetes, Type 1[Title/Abstract])) OR (Diabetes Mellitus, Type I[Title/Abstract])) OR (Autoimmune Diabetes[Title/Abstract]) | 538,806 |
| #2 | "Dietary Carbohydrates"[Mesh Terms] OR "Nutrition Therapy"[Mesh Terms] OR "Diet Therapy"[Mesh Terms]OR Carbohydrate counting[Title/Abstract] OR Carbohydrate[Title/Abstract] OR carb counting[Title/Abstract] OR carb-counting[Title/Abstract] OR Carbohydrate exchange[Title/Abstract] OR Carbohydrate portion[Title/Abstract] OR Insulin to carbohydrate ratio[Title/Abstract] | 332,688 |
| #3 | "Augmented Reality"[Mesh]) OR (Augmented Realities[Title/Abstract])) OR (Realities, Augmented[Title/Abstract])) OR (Reality, Augmented[Title/Abstract])) OR (Mixed Reality[Title/Abstract])) OR (Mixed Realities[Title/Abstract])) OR (Realities, Mixed[Title/Abstract])) OR (Reality, Mixed[Title/Abstract]) | 4,404 |
| #4 | ("Virtual Reality"[Mesh]) OR (Reality, Virtual[Title/Abstract]) | 7,438 |
| #5 | "Artificial Intelligence"[Mesh Terms] OR ntelligence, Artificial[Title/Abstract] OR Computational Intelligence[Title/Abstract] OR Intelligence, Computational[Title/Abstract] OR Intelligence, Machine[Title/Abstract] OR Machine Intelligence[Title/Abstract] OR Computer Reasoning[Title/Abstract] OR Reasoning, Computer[Title/Abstract] OR AI Artificial Intelligence[Title/Abstract] OR Computer Vision System[Title/Abstract] OR System, Computer Vision[Title/Abstract] OR Vision System, Computer[Title/Abstract] OR Knowledge Acquisition Computer[Title/Abstract] OR Acquisition, Knowledge Computer[Title/Abstract] OR Knowledge Representation Computer[Title/Abstract] OR Representation, Knowledge Computer[Title/Abstract] | 210,141 |
| #6 | "Mobile Applications"[Mesh Terms] OR Application, Mobile[Title/Abstract] OR Applications, Mobile[Title/Abstract] OR Mobile Application[Title/Abstract] OR Mobile Apps[Title/Abstract] OR App, Mobile[Title/Abstract] OR Apps, Mobile[Title/Abstract] OR Mobile App[Title/Abstract] OR Portable Software Apps[Title/Abstract] OR App, Portable Software[Title/Abstract] OR Portable Software App[Title/Abstract] OR Software App, Portable[Title/Abstract] OR Portable Software Applications[Title/Abstract] OR Application, Portable Software[Title/Abstract] OR Portable Software Application[Title/Abstract] OR Software Application, Portable[Title/Abstract] OR Smartphone Apps[Title/Abstract] OR App, Smartphone[Title/Abstract] OR Apps, Smartphone[Title/Abstract] OR Smartphone App[Title/Abstract] OR Portable Electronic Apps[Title/Abstract] OR Electronic App, Portable[Title/Abstract] OR App, Portable Electronic[Title/Abstract] OR Portable Electronic App[Title/Abstract] OR Portable Electronic Applications[Title/Abstract] OR Application, Portable Electronic[Title/Abstract] OR Electronic Application, Portable[Title/Abstract] OR Portable Electronic Application[Title/Abstract] | 21,499 |
| #7 | "Software"[Mesh Terms] OR Computer Software[Title/Abstract] OR Software, Computer[Title/Abstract] OR Open Source Software[Title/Abstract] OR Open Source Softwares[Title/Abstract] OR Software, Open Source[Title/Abstract] OR Softwares, Open Source[Title/Abstract] OR Source Software, Open[Title/Abstract] OR Source Softwares, Open[Title/Abstract] OR Computer Programs[Title/Abstract] OR Computer Program[Title/Abstract] OR Program, Computer[Title/Abstract] OR Programs, Computer[Title/Abstract] OR Software Tools[Title/Abstract] OR Software Tool[Title/Abstract] OR Tool, Software[Title/Abstract] OR Tools, Software[Title/Abstract] OR Software Engineering[Title/Abstract] OR Engineering, Software[Title/Abstract] OR Computer Applications Software[Title/Abstract] OR Applications Software, Computer[Title/Abstract] OR Applications Softwares, Computer[Title/Abstract] OR Computer Applications Softwares[Title/Abstract] OR Software, Computer Applications[Title/Abstract] OR Softwares, Computer Applications[Title/Abstract] OR Computer Software Applications[Title/Abstract] OR Application, Computer Software[Title/Abstract] OR Computer Software Application[Title/Abstract] OR Applications, Computer Software[Title/Abstract] OR Software Application, Computer[Title/Abstract] OR Software Applications, Computer[Title/Abstract] OR Computer Programs[Title/Abstract] OR Programming[Title/Abstract] | 262,894 |
| #8 | "Telemedicine"[Mesh] OR Tele-Referral[Title/Abstract] OR Tele Referral[Title/Abstract] OR Tele-Referrals[Title/Abstract] OR Virtual Medicine[Title/Abstract] OR Medicine, Virtual[Title/Abstract] OR Tele-Intensive Care[Title/Abstract] OR Tele-Intensive Care[Title/Abstract] OR Tele-Intensive Care[Title/Abstract] OR Tele ICU[Title/Abstract] OR Mobile Health[Title/Abstract] OR Mobile Health[Title/Abstract] OR mHealth[Title/Abstract] OR Telehealth[Title/Abstract] OR Telehealth[Title/Abstract] OR telemedicine system[Title/Abstract] OR telemedicine systems[Title/Abstract] | 67,765 |
| #9 | "Internet"[Mesh] OR World Wide Web[Title/Abstract] OR Web, World Wide[Title/Abstract] OR Wide Web, World[Title/Abstract] OR Cyberspace[Title/Abstract] OR Cyber Space[Title/Abstract] OR Computer Systems[Title/Abstract] OR Computer Communication Networks[Title/Abstract] | 106,894 |
| #10 | "Digital Technology"[Mesh] OR Digital Technologies[Title/Abstract] OR Technologies, Digital[Title/Abstract] OR Technology, Digital[Title/Abstract] OR Digital Electronics[Title/Abstract] OR Electronics, Digital[Title/Abstract] | 4,554 |
| #11 | "Computers"[Mesh] OR Computer[Title/Abstract] OR Calculators, Programmable[Title/Abstract] OR Calculator, Programmable[Title/Abstract] OR Programmable Calculator[Title/Abstract] OR Programmable Calculators[Title/Abstract] OR Hardware, Computer[Title/Abstract] OR Computer Hardware[Title/Abstract] OR Computer Hardware[Title/Abstract] OR Computer, Digital[Title/Abstract] OR Digital Computer[Title/Abstract] | 333,663 |
| #12 | "Text Messaging"[Mesh] OR Messaging, Text[Title/Abstract] OR Texting[Title/Abstract] OR Short Message Service[Title/Abstract] OR Text Messages[Title/Abstract] OR Message, Text[Title/Abstract] OR Messages, Text[Title/Abstract] OR Text Message[Title/Abstract] OR Short message[Title/Abstract] OR Shor messages[Title/Abstract] OR SMS[Title/Abstract] | 16,131 |
| #13 | "Video-Audio Media" [Publication Type] OR "Videotape Recording"[Mesh] OR Recording, Videotape[Title/Abstract] OR Recording, Videotape[Title/Abstract] OR Recording, Videotape[Title/Abstract] OR Tape Recording, Video[Title/Abstract] OR Tape Recording, Video[Title/Abstract] OR Recordings, Video Tape[Title/Abstract] OR Tape Recordings, Video[Title/Abstract] OR Video Tape Recording[Title/Abstract] OR Video Tape Recordings[Title/Abstract] OR Videotapes[Title/Abstract] OR Videotape[Title/Abstract] OR viedo[Title/Abstract] OR videoes[Title/Abstract] | 59,626 |
| #14 | "Glycated Hemoglobin"[Mesh Terms] OR "Hemoglobin A"[Mesh Terms] OR Hemoglobin, Glycated[Title/Abstract] OR Glycohemoglobin[Title/Abstract] OR Glycohemoglobins[Title/Abstract] OR Glycated Hemoglobins[Title/Abstract] OR Hemoglobins, Glycated[Title/Abstract] OR Hemoglobin, Glycosylated[Title/Abstract] OR Glycosylated Hemoglobin[Title/Abstract] OR Glycated Hemoglobin A1c[Title/Abstract] OR Hemoglobin A1c, Glycated[Title/Abstract] OR Glycosylated Hemoglobin A1c[Title/Abstract] OR Hemoglobin A1c, Glycosylated[Title/Abstract] OR Hb A1a-2[Title/Abstract] OR Hemoglobin, Glycated A1a-2[Title/Abstract] OR A1a-2 Hemoglobin, Glycated[Title/Abstract] OR Glycated A1a-2 Hemoglobin[Title/Abstract] OR Hemoglobin, Glycated A1a 2[Title/Abstract] OR Glycated Hemoglobin A[Title/Abstract] OR Hemoglobin A, Glycated[Title/Abstract] OR Hb A1a+b[Title/Abstract] OR Hb A1c[Title/Abstract] OR HbA1[Title/Abstract] OR Glycosylated Hemoglobin A[Title/Abstract] OR Hemoglobin A, Glycosylated[Title/Abstract] OR Hb A1[Title/Abstract] OR Glycohemoglobin A[Title/Abstract] OR Hemoglobin A1[Title/Abstract]OR Hemoglobin, Glycosylated A1a-1[Title/Abstract] OR A1a-1 Hemoglobin, Glycosylated[Title/Abstract] OR Glycosylated A1a-1 Hemoglobin[Title/Abstract] OR Hemoglobin, Glycosylated A1a 1[Title/Abstract] OR Hb A1a-1[Title/Abstract] OR Hemoglobin, Glycated A1b[Title/Abstract] OR A1b Hemoglobin, Glycated[Title/Abstract] OR Glycated A1b Hemoglobin[Title/Abstract] OR Hb A1b[Title/Abstract] OR Hemoglobin, Glycosylated A1b[Title/Abstract] OR A1b Hemoglobin, Glycosylated[Title/Abstract] OR Glycosylated A1b Hemoglobin[Title/Abstract] OR Fructated Hemoglobins[Title/Abstract] OR Hemoglobins, Fructated[Title/Abstract] | 57,346 |
| #15 | #3 OR #4 OR #5 OR #6 OR #7 OR #8 OR #9 OR #10 OR #11 OR #12 OR #13 | 931,996 |
| #16 | #1 AND #2 AND #14 AND #15 | 71 |
