# Supplementary material for: Effectiveness of Telemedicine-Delivered Carbohydrate-Counting Interventions in Patients With Type 1 Diabetes: Systematic Review and Meta-Analysis
Source: J Med Internet Res. 2025 Apr 10;27:e59579. doi: 10.2196/59579 (PMC12022529; doi:10.2196/59579)
Supplement: Multimedia Appendix 2 [file jmir_v27i1e59579_app2.docx]

Table 2. Web Of Science Core Collection Search Trail (Search updated 26/09/2024)

| Search # | MeSH Terms and Key Words | Articles Revealed |
| --- | --- | --- |
| #1 | Diabetes Mellitus (Topic) OR Diabetes Mellitus, Type 1 (Topic) OR Diet, Diabetic (Topic) OR Diabetes Mellitus, Insulin-Dependent (Topic) OR Type 1 Diabetes Mellitus (Topic) OR Diabetes, Type 1 (Topic) OR Diabetes Mellitus, Type I (Topic) OR Autoimmune Diabetes (Topic) | 729,326 |
| #2 | Carbohydrate counting (Topic) OR Carbohydrate (Topic) OR carb counting (Topic) OR carb-counting (Topic) OR Carbohydrate exchange (Topic) OR Carbohydrate portion (Topic) OR Insulin to carbohydrate ratio (Topic) OR Dietary Carbohydrates (Topic) OR Nutrition Therapy (Topic) OR Diet Therapy (Topic) | 1,294,263 |
| #3 | Telemedicine (Topic) OR Tele-Referral (Topic) OR Tele Referra (Topic) OR Tele-Referrals (Topic) OR Virtual Medicine (Topic) OR Medicine, Virtual (Topic) OR Tele-Intensive Care (Topic) OR Tele-Intensive Care (Topic) OR Tele-Intensive Care (Topic) OR Tele ICU (Topic) OR Mobile Health (Topic) OR Mobile Health (Topic) OR mHealth (Topic) OR Telehealth (Topic) OR Telehealth (Topic) OR telemedicine system (Topic) | 174,674 |
| #4 | Virtual Reality (Topic) OR Reality,Virtual (Topic) | 109,556 |
| #5 | Augmented Reality (Topic) OR Augmented Realities (Topic) OR Realities, Augmented (Topic) OR Reality, Augmented (Topic) OR Mixed Reality OR Mixed Realities (Topic) OR Realities, Mixed (Topic) OR Reality, Mixed (Topic) | 48,546 |
| #6 | Internet (Topic) OR World Wide Web (Topic) OR Web, World Wide (Topic) OR Wide Web, World (Topic) OR Cyberspace (Topic) OR Cyber Space (Topic) OR Computer Systems (Topic) OR Computer Communication Networks (Topic) | 3,249,561 |
| #7 | Digital Technology (Topic) OR Digital Technologies (Topic) OR Technologies, Digital (Topic) OR Technology, Digita (Topic) OR Digital Electronics (Topic) OR Electronics, Digital (Topic) | 336,565 |
| #8 | Computers (Topic) OR Computer (Topic) OR Calculators, Programmable (Topic) OR Calculator, Programmable (Topic) OR Programmable Calculator (Topic) OR Programmable Calculators (Topic) OR Hardware, Computer (Topic) OR Computer Hardware (Topic) OR Computer Hardware (Topic) OR Computer, Digital (Topic) OR Digital Computer (Topic) | 4,865,529 |
| #9 | Text Messaging (Topic) OR Messaging, Text (Topic) OR Texting (Topic) OR Short Message Service (Topic) OR Text Messages (Topic) OR Message, Text (Topic) OR Messages, Text (Topic) OR Text Message (Topic) OR Short message (Topic) OR Shor messages (Topic) OR SMS (Topic) | 56,132 |
| #10 | Video-Audio Media (Topic) OR Videotape Recording (Topic) OR Recording, Videotape (Topic) OR Recording, Videotape (Topic) OR Recording, Videotape (Topic) OR Tape Recording, Video (Topic) OR Tape Recording, Video (Topic) OR Recordings, Video Tape (Topic) OR Tape Recordings, Video (Topic) OR Video Tape Recording (Topic) OR Video Tape Recordings (Topic) OR Videotapes (Topic) OR Videotape (Topic) OR viedo (Topic) OR videoes (Topic) | 22,827 |
| #11 | Artificial Intelligence (Topic) OR Intelligence, Artificial (Topic) OR Computational Intelligence (Topic) OR Intelligence, Computational (Topic) OR Intelligence, Machine (Topic) OR Machine Intelligence (Topic) OR Computer Reasoning (Topic) OR Reasoning, Computer (Topic) OR AI (Artificial Intelligence) (Topic) OR Computer Vision System (Topic) OR System, Computer Vision (Topic) OR Vision System, Computer (Topic) OR Knowledge Acquisition Computer (Topic) OR Acquisition, Knowledge Computer (Topic) OR Knowledge Representation Compute (Topic) OR Representation, Knowledge Computer (Topic) | 1,149,501 |
| #12 | Mobile Applications (Topic) OR Application, Mobile (Topic) OR Applications, Mobile (Topic) OR Mobile Application (Topic) OR Mobile Apps (Topic) OR App, Mobile (Topic) OR Apps, Mobile (Topic) or Mobile App (Topic) OR Portable Software Apps (Topic) OR App, Portable Software (Topic) or Portable Software App (Topic) OR Software App, Portable (Topic) OR Portable Software Applications (Topic) OR Application, Portable Software (Topic) OR Portable Software Application (Topic) OR Software Application, Portable (Topic) OR Smartphone Apps (Topic) OR App, Smartphone (Topic) OR Apps, Smartphone (Topic) OR Smartphone App (Topic) OR Portable Electronic Apps (Topic) OR Electronic App, Portable (Topic) OR App, Portable Electronic (Topic) OR Portable Electronic App (Topic) OR Portable Electronic Applications (Topic) OR Application, Portable Electronic (Topic) OR Electronic Application, Portable (Topic) OR Portable Electronic Application (Topic) | 270,467 |
| #13 | Program (Topic) OR Program, Computer (Topic) OR Programs, Computer (Topic) OR Software Tools (Topic) OR Software Tool (Topic) OR Tool, Software (Topic) OR Tools, Software (Topic) OR Software Engineering (Topic) OR Engineering, Software (Topic) OR Computer Applications Software (Topic) OR Applications Software, Computer (Topic) OR Applications Softwares, Computer (Topic) OR Computer Applications Softwares (Topic) OR Software, Computer Applications (Topic) OR Softwares, Computer Applications (Topic) OR Computer Software Applications (Topic) OR Application, Computer Software (Topic) OR Computer Software Application (Topic) OR Applications, Computer Software (Topic) OR Software Application, Computer (Topic) OR Software Applications, Computer (Topic) OR Computer Programs (Topic) OR Programming (Topic) | 3,186,443 |
| #14 | Glycated Hemoglobin (Topic) OR Hemoglobin A (Topic) OR Hemoglobin, Glycated (Topic) OR Glycohemoglobin (Topic) OR Glycated Hemoglobins (Topic) OR Glycohemoglobins (Topic) or Hemoglobins, Glycated (Topic) OR Hemoglobin, Glycosylated (Topic) OR Glycosylated Hemoglobin (Topic) OR Hemoglobin A1c, Glycosylated (Topic) OR Hb A1a-2 (Topic) OR Hemoglobin, Glycated A1a-2 (Topic) OR A1a-2 Hemoglobin, Glycated (Topic) OR Glycated A1a-2 Hemoglobin (Topic) OR Hemoglobin, Glycated A1a 2 (Topic) OR Glycated Hemoglobin A (Topic) OR Hemoglobin A, Glycated (Topic) OR Hb A1a+b (Topic) OR Hb A1c (Topic) or HbA1 (Topic) OR Glycosylated Hemoglobin A (Topic) OR Hemoglobin A, Glycosylated (Topic) OR Hb A1 (Topic) OR Glycohemoglobin A (Topic) OR Hemoglobin A (Topic) OR Hemoglobin, Glycosylated A1a-1 (Topic) OR A1a-1 Hemoglobin, Glycosylated (Topic) OR Glycosylated A1a-1 Hemoglobin (Topic) OR Hemoglobin, Glycosylated A1a 1 (Topic) OR Hb A1a-1 (Topic) OR Hemoglobin, Glycated A1b (Topic) OR A1b Hemoglobin, Glycated (Topic) OR Glycated A1b Hemoglobin (Topic) OR Hb A1b (Topic) OR Hemoglobin, Glycosylated A1b (Topic) OR A1b Hemoglobin, Glycosylated (Topic) OR Glycosylated A1b Hemoglobin (Topic) OR Fructated Hemoglobins (Topic) OR Hemoglobins, Fructated (Topic) | 212,700 |
| #15 | #3 OR #4 OR #5 OR #6 OR #7 OR #8 OR #9 OR #10 OR #11 OR #12 OR #13 | 8,284,885 |
| #16 | #1 AND #2 AND #13 AND #14 | 3015 |
