# Supplementary material for: Effectiveness of Telemedicine-Delivered Carbohydrate-Counting Interventions in Patients With Type 1 Diabetes: Systematic Review and Meta-Analysis
Source: J Med Internet Res. 2025 Apr 10;27:e59579. doi: 10.2196/59579 (PMC12022529; doi:10.2196/59579)
Supplement: Multimedia Appendix 3 [file jmir_v27i1e59579_app3.docx]

Table 3. Embase Search Trail (Search updated 26/09/2024)

| Search # | MeSH Terms and Key Words | Articles Revealed |
| --- | --- | --- |
| #1 | 'diabetes mellitus'/exp OR 'diabetes mellitus':ab,ti OR diabetes:ab,ti | 1,538,827 |
| #2 | 'carbohydrate intake'/exp OR 'carbohydrate intake':ab,ti OR 'saccharide intake':ab,ti OR 'carbohydrate countin':ab,ti OR 'carbohydrate exchange':ab,ti OR 'diet therapy':ab,ti | 42,941 |
| #3 | 'virtual reality'/exp OR 'virtual reality':ab,ti | 36,103 |
| #4 | 'augmented reality'/exp OR 'augmented reality':ab,ti | 6,491 |
| #5 | 'telemedicine'/exp OR telemedicine:ab,ti OR 'tele medicine':ab,ti OR 'virtual medicine':ab,ti | 83,700 |
| #6 | 'software'/exp OR software:ab,ti OR 'computer code':ab,ti OR 'computer program':ab,ti OR 'computer programme':ab,ti OR 'program, computer':ab,ti OR 'programme, computer':ab,ti | 568,874 |
| #7 | 'mobile application'/exp OR 'mobile application':ab,ti OR 'mobile app':ab,ti OR 'mobile apps':ab,ti | 31,556 |
| #8 | 'artificial intelligence'/exp OR 'artificial intelligence':ab,ti OR 'machine intelligenceartificial':ab,ti OR intelligence:ab,ti OR 'computer vision system':ab,ti | 169,875 |
| #9 | 'internet'/exp OR internet:ab,ti OR 'world wide web':ab,ti OR 'internet connection':ab,ti | 173,479 |
| #10 | 'text messaging'/exp OR 'text messaging':ab,ti OR texting:ab,ti OR 'short message':ab,ti OR 'short messages':ab,ti OR sms:ab,ti OR 'messaging, text':ab,ti OR 'short message service':ab,ti | 20,097 |
| #11 | 'video recording'/exp OR 'video recording':ab,ti OR video:ab,ti | 227,068 |
| #12 | 'digital technology'/exp OR 'digital technology':ab,ti OR 'technologies, digital':ab,ti OR 'technology, digital':ab,ti OR 'digital electronics':ab,ti OR 'electronics, digital':ab,ti | 9,345 |
| #13 | 'glycated hemoglobin'/exp OR glycohaemoglobin:ab,ti OR 'glycosyl haemoglobin':ab,ti OR 'glycosylated haemoglobin':ab,ti OR glycosylhaemoglobin:ab,ti OR glycosylhemoglobin:ab,ti OR 'glycosylised haemoglobin':ab,ti OR 'glycosylized hemoglobin':ab,ti OR 'haemoglobin a, glycosylated':ab,ti OR 'haemoglobin a 1':ab,ti OR 'haemoglobin ai':ab,ti OR 'haemoglobin alpha 1':ab,ti OR 'haemoglobin glycoside':ab,ti OR 'haemoglobin glycosylation':ab,ti OR 'hemoglobin a, glycosylate':ab,ti OR 'hemoglobin glycoside':ab,ti OR 'glycated hemoglobin':ab,ti | 196,272 |
| #14 | #3 OR #4 OR #5 OR #6 OR #7 OR #8 OR #9 OR #10 OR #11 OR #12 | 1,477,398 |
| #15 | #1 AND #2 AND #13AND #14 | 155 |
