# Supplementary material for: Effectiveness of Telemedicine-Delivered Carbohydrate-Counting Interventions in Patients With Type 1 Diabetes: Systematic Review and Meta-Analysis
Source: J Med Internet Res. 2025 Apr 10;27:e59579. doi: 10.2196/59579 (PMC12022529; doi:10.2196/59579)
Supplement: Multimedia Appendix 4 [file jmir_v27i1e59579_app4.docx]

Table 4. CINAHL Search Trail (Search updated 26/09/2024)

| Search # | MeSH Terms and Key Words | Articles Revealed |
| --- | --- | --- |
| #1 | (MH "Diabetes Mellitus+") OR (MH "Diabetes Mellitus, Type 2") OR (MM "Diabetes Mellitus, Type 1+") OR (MM "Diabetes Mellitus, Gestational") OR (MM "Diabetes Education") | 195,864 |
| #2 | (MM "Dietary Carbohydrates+") OR (MM "Carbohydrates+") | 62,265 |
| #3 | (MM "Telemedicine+") OR (MM "Telehealth+") OR (MM "Telerehabilitation") OR (MM "Teleradiology") OR (MM "Telepathology") OR (MM "Remote Consultation") OR (MM "Telenutrition") OR (MM "Communications Media+") OR (MM "Telecommunications+") OR (MM "Information Science+") OR (MM "Teledentistry") OR (MM "Telenursing") | 647,996 |
| #4 | (MM "Virtual Reality+") | 5,040 |
| #5 | (MM "Augmented Reality") | 525 |
| #6 | (MH "Artificial Intelligence") OR (MH "Virtual Reality") OR (MH "Video Games") OR (MH "Software Design") OR (MH "Image Processing, Computer Assisted") | 47,108 |
| #7 | (MM "Mobile Applications") OR (MM "Multimedia") OR (MM "Web Browsers") OR (MM "Operating Systems") OR (MM "Software+") OR (MM "Communications Software+") OR (MM "Computer Systems+") | 38,655 |
| #8 | (MM "World Wide Web+") OR (MM "Internet+") OR (MM "World Wide Web Applications+") | 65,518 |
| #9 | (MM "Digital Technology+") OR (MM "Information Technology+") OR (MM "Information Science+") | 647,984 |
| #10 | (MM "Instant Messaging") OR (MM "Text Messaging") | 3,042 |
| #11 | (MM "Video Games+") OR (MM "Digital Versatile Disc") OR (MM "Videorecording+") | 11,510 |
| #12 | (MM "Glycated Hemoglobin") OR (MM "Hemoglobins+") OR (MM "Hemoglobin A+") | 7,968 |
| #13 | #3 OR #4 OR #5 OR #6 OR #7 OR #8 OR #9 OR #10 OR #11 | 663,312 |
| #14 | #1 AND #2 AND #12 AND #13 | 131 |
