# Supplementary material for: Effectiveness of Telemedicine-Delivered Carbohydrate-Counting Interventions in Patients With Type 1 Diabetes: Systematic Review and Meta-Analysis
Source: J Med Internet Res. 2025 Apr 10;27:e59579. doi: 10.2196/59579 (PMC12022529; doi:10.2196/59579)
Supplement: Multimedia Appendix 5 [file jmir_v27i1e59579_app5.docx]

Table 5. Cochrane Library Search Trail (Search updated 26/09/2024)

| Search # | MeSH Terms and Key Words | Articles Revealed |
| --- | --- | --- |
| #1 | MeSH descriptor: [Diabetes Mellitus] explode all trees OR Diabetes Mellitus, Type 1 OR Diet, Diabetic | 73634 |
| #2 | MeSH descriptor: [Dietary Carbohydrates] explode all trees OR Dietary Carbohydrate OR Carbohydrate OR Carbohydrate counting OR Carbohydrate exchange | 21822 |
| #3 | MeSH descriptor: [Virtual Reality] explode all trees OR **Virtual Reality, Instructional** OR  **Virtual Realities, Instructional** OR **Instructional Virtual Reality** OR **Reality, Instructional Virtua** OR **Educational Virtual Reality** OR **Reality, Educational Virtual** OR **Virtual Reality, Educational** OR **Virtual Realities, Educational; Educational Virtual Realities** OR **Instructional Virtual Realities** OR **Realities, Instructional Virtual** OR **Reality, Virtual** | 7546 |
| #4 | MeSH descriptor: [Augmented Reality] explode all trees OR **Mixed Reality** OR **Augmented Realities** OR **Reality, Mixed** OR **Reality, Augmented** OR **Realities, Mixed** OR **Realities, Augmented** OR **Mixed Realities** | 1272 |
| #5 | MeSH descriptor: [Artificial Intelligence] explode all trees OR Machine Intelligence OR Intelligence OR Vision Systems, Computer | 9804 |
| #6 | MeSH descriptor: [Mobile Applications] explode all trees OR Software App, Portable OR Software Application, Portable OR Smartphone APP OR Electronic Application, Portable | 5130 |
| #7 | MeSH descriptor: [Software] explode all trees OR Software Tool OR Program,computer OR Software; Application OR Open Source Software | 33699 |
| #8 | MeSH descriptor: [Telemedicine] explode all trees OR Tele-Referrals OR mHealth OR eHealth OR Mobile Health OR Virtual Medicine OR Health, Mobile OR Tele Intensive Care OR Telehealth OR Tele-Intensive Care | 21386 |
| #9 | MeSH descriptor: [Internet] explode all trees OR Wide Web, World OR World Wide Web OR Web, World Wide OR Cyber Space OR Cyberspace | 6970 |
| #10 | MeSH descriptor: [Digital Technology] explode all trees OR Electronics, Digital OR Digital Electronics OR Digital Technologies OR Technology, Digital OR Technologies, Digital | 2303 |
| #11 | MeSH descriptor: [Computers] explode all trees OR Computer OR Computer, Digital OR Digital Computer OR Digital Computers OR Computers, Digital OR Hardware, Computer OR Computer Hardware OR Programmable Calculators OR Calculator, Programmable OR Calculators, Programmable OR Programmable Calculator | 237784 |
| #12 | MeSH descriptor: [Text Messaging] explode all trees OR Messaging, Text OR Texting OR Textings OR Text Message OR Messages, Text OR Message, Text OR Text Messages OR Short Message Service OR Short message OR Shor messages OR SMS | 260316 |
| #13 | MeSH descriptor: [Video-Audio Media] explode all trees OR Audiovisual Medi OR Audio-Visual Media OR viedo OR videoes | 4 |
| #14 | (Glycated Hemoglobin):ti,ab,kw OR Hemoglobin A1c OR Glycated Hemoglobin A1c OR Glycosylated Hemoglobin A1c OR Hemoglobin A1c | 18699 |
| #15 | #3 OR #4 OR #5 OR #6 OR #7 OR #8 OR #9 OR #10 OR #11 OR #12 OR #13 | 480498 |
| #16 | #1 AND #2 AND #13 AND #14 | 240 |
